# The Liver and Kidneys mediate clearance of cardiac troponin in the rat

**DOI:** 10.1038/s41598-020-63744-8

**Published:** 2020-04-22

**Authors:** Aida Muslimovic, Vincent Fridén, Olav Tenstad, Karin Starnberg, Susanne Nyström, Emelie Wesén, Elin K. Esbjörner, Kristoffer Granholm, Bertil Lindahl, Ola Hammarsten

**Affiliations:** 1 Institute of Biomedicine, Department of Laboratory Medicine, Sahlgrenska University Hospital, Gothenburg University, Gothenburg, Sweden; 2 Department of Biomedicine, University of Bergen, Bergen, Norway; 3 Department of Biology and Biotechnology, Biology and Biological Engineering, Chemical Biology, Chalmers University of Technology, Gothenburg, Sweden; 4 Department of Medical Sciences, Cardiology and Uppsala Clinical Research Center, Uppsala University, Uppsala, Sweden

**Keywords:** Diagnostic markers, Cardiovascular biology, Cardiovascular diseases

## Abstract

Cardiac-specific troponins (cTn), troponin T (cTnT) and troponin I (cTnI) are diagnostic biomarkers when myocardial infarction is suspected. Despite its clinical importance it is still not known how cTn is cleared once it is released from damaged cardiac cells. The aim of this study was to examine the clearance of cTn in the rat. A cTn preparation from pig heart was labeled with fluorescent dye or fluorine 18 (^18^ F). The accumulation of the fluorescence signal using organ extracts, or the 18 F signal using positron emission tomography (PET) was examined after a tail vein injection. The endocytosis of fluorescently labeled cTn was studied using a mouse hepatoma cell line. Close to 99% of the cTnT and cTnI measured with clinical immunoassays were cleared from the circulation two hours after a tail vein injection. The fluorescence signal from the fluorescently labeled cTn preparation and the radioactivity from the 18F-labeled cTn preparation mainly accumulated in the liver and kidneys. The fluorescently labeled cTn preparation was efficiently endocytosed by mouse hepatoma cells. In conclusion, we find that the liver and the kidneys are responsible for the clearance of cTn from plasma in the rat.

## Introduction

Cardiac troponin (cTn) is a ternary complex consisting of three distinct subunits: cardiac troponin T (cTnT), cardiac troponin I (cTnI) and cardiac troponin C (cTnC). cTn binds thin filaments within the cardiomyocyte sarcomere and, with tropomyosin, makes muscular contraction dependent on calcium ions^1^. Both cTnT and cTnI are released into the circulation following cardiac damage^2^ and are the preferred cardiac biomarkers when myocardial infarction (MI) is suspected^3^. The use of cTn assays with low diagnostic cutoffs improve diagnostic accuracy for MI on the emergency departments^4,5^ and reduce hospital spending^6^. However, low diagnostic cutoffs have been accompanied by an accumulation of patients presenting with stable cTn levels above the accepted cutoff point without MI^7,8^. A third of older emergency department patients without MI on emergency departments have stable cTn elevations^7^, often for unknown reasons^9^, and are generally admitted to have their workup done^6,10^.

Even if MI can be excluded during the hospital stay, patients with stable cTn elevations still constitute a significant health care problem, as a stable cTn elevation is a strong risk factor for the development of heart disease and death^11–14^.

The reason behind stable cTn elevations, where sample-to-sample variation is often 10%^7^ but can be as large as 40–50%^15^, is still unclear but is typically found at old age, in patients with decreased renal function or with comorbidities^10^. It is possible that, in addition to necrosis, cTnT and cTnI are also released from living cardiomyocytes under ischemic stress^16^ and possibly from normal cardiomyocytes as well^17^. To understand the possible mechanisms that link stable cTn elevations to mortality, we need to know how cTn is released and cleared from the circulation^10,18,19^.

Surprisingly little is known about how cTn and other cardiac damage biomarkers, such as myoglobin, are cleared from the circulation. We know from our previous studies that cTnT and cTnI are cleared, in part, by the kidneys in rats and humans^2,20^. This could be a possible mechanism behind the stable cTn elevations in patients^10,18,19^ and rats^21^ with decreased kidney function. However, we also observe rapid clearance of cTnT and cTnI in rats without renal function^2,20^. Apparently, there is also prominent extrarenal clearance of cTnT. To examine this in greater detail, we labeled a pig cTn complex and examined its clearance in the rat.

## Methods

### Animals

Male Wistar Kyoto rats (Harlan, Horst, The Netherlands) kept on standard fodder and with free access to water were used, unless otherwise stated. Anesthesia was induced and maintained by inhalation of isoflurane (Isobavet, Schering-Plough Animal Health, Buckinghamshire, UK, 4.5% induction and 3.2–3.9% maintenance), using the Univentor 400 anesthesia unit (AgnTho’s AB, Lidingö, Sweden). Anesthetized animals from injection experiments were euthanized after cardiac puncture, the hearts were dissected, rinsed in ice-cold PBS and sliced before freezing in liquid nitrogen for storage at −80°. Pig cardiac tissue was kindly provided by the Experimental Biomedicine (EBM) Core facility at Gothenburg University. The hearts were frozen in liquid nitrogen before storage at −80°. Both pig and rat hearts were used for preparations of cTn. All protocols and procedures involving animal experiments were in accordance with the guidelines in Directive 2010/63/EU of the European Parliament and of the Council on the protection of animals used for scientific purposes, and approved by the Regional Board for Ethical Review of research projects using animals in Gothenburg, appointed by the Swedish Ministry of Agriculture (Ethical Approval # 282–2012).

### Purification of cTn complexes from pig and rat hearts

**Figure 1:**
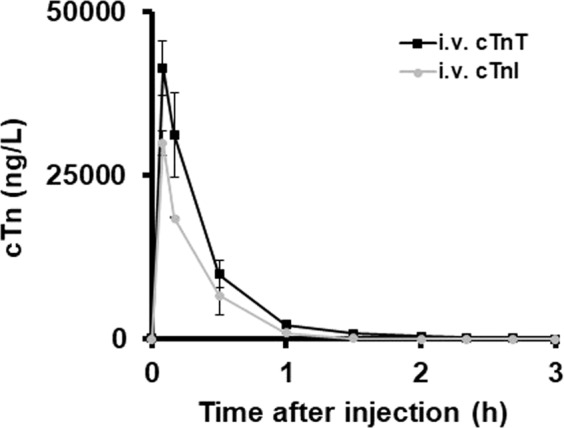
Kinetics of cTnT and cTnI clearance after a jugular vein injection of purified pig cTn complex. Mean values from three rats. Error bars represent ±1 SD.

Both pig and rat hearts were used for purification of cTn complexes. The amount of purified cTn obtained from rat hearts were limited. We therefore prepared cTn from pig hearts that were available in substantially higher quantity. The cTn complex was essentially prepared as described^22^, using 700 g of frozen left ventricular cardiac pig tissue or 8 g of left ventricular cardiac rat tissue. The method below describes the cTn preparation from left ventricular cardiac pig tissue. However, the same procedure was used for the cardiac rat tissue that was used in the animal experiments. The diethyl ether powder was extracted twice for 1 h at a time with 400 mL phosphate-buffered saline, PBS, supplemented with 1 M NaCl, 1 mM CaCl_2_ and 1 mM Dithiothreitol, DTT, and protease inhibitors (1x cOmplete™, EDTA-free Protease Inhibitor Cocktail) (Roche). The extract was fractionated by ammonium sulphate precipitation as described. The 43.5% ammonium sulphate pellet was dissolved in a total of 110 mL Buffer P (0.1 M Tris-HCl pH 7.5, 0.1 M NaCl, 0.1 m M CaCl_2_), cleared by centrifugation and loaded on a 10 mL MMC column (GE Health Care) equilibrated with Buffer P, washed with five column volumes of PBS supplemented with 1 M NaCl, followed by five column volumes of MQ water and finally developed with a 100 mL gradient using 50 mL 1 M NaCl, 0.1 M NaOH and 2 mL fractions were collected. The fractions were rapidly supplemented with 200 µl Tris-HCl, pH 7.5, 1 mM DTT to normalize the pH level, and stored at −20 °C. The final yield from the 700 g of heart tissue was 23 mg cTn, measured with the Bicinchoninic Acid Kit for Protein Determination (Sigma Aldrich) with bovine serum albumin as standard. The presence of cTnT and cTnI was confirmed using the Abbott STAT high-sensitivity cTnI assay and the Roche high-sensitivity cTnT assay. The presence of troponin C was inferred from overloaded SDS gels (Supplementary Fig. 2) but not directly detected or measured. cTnT and cTnI co-eluted just before bovine serum albumin (60 kDa) in size exclusion chromatography on a 40 mL Sephacryl S-300 as measured by the Roche hs-cTnT method on Cobas and the Abbott hs-cTnT method on Architect, indicating a molecular complex weight of roughly 80 kDa, as previously reported^22,23^. There was no elution of cTnI at 24 kDa in the chromatogram, indicating that no free cTnI was present in the preparation (data not shown). In addition, the cTnT and cTnI levels were the same in blood samples collected from rats injected with the pig cTn preparation (Fig. 1), indicating that most of the cTnT and cTnI in the pig cTn preparation was present in a cTnT/cTnI complex. However, as it has been shown that monomeric cTnT and cTnT/cTnI/cTnC complexes co-elute on gel filtration chromatography^23^, we cannot determine whether all of the cTnT was present in a complex with cTnI. Indeed, some of the cTn preparation precipitated if kept at concentrations >2 g/L in low-salt buffers. SDS gel analysis of this precipitate indicated that it was mostly cTnT that precipitated. cTnT is known to be unstable if not in a complex with cTnI^22^. For that reason, as described below, cTn preparations were kept in high-salt buffers until they were diluted > 100-fold in physiological buffers and centrifuged at 13 000xG to remove any precipitate before injection in animals.

The undiluted peak fractions with a protein concentration of 2 mg/mL had a tendency to precipitate at low ionic strength. For that reason, the cTn fractions were stored and labeled with Alexa-NHS dyes in buffers containing 1 M NaCl as described in next section. Prior to injection in the animals, the cTn preparations were diluted to 100-fold lower concentrations (<0.1 mg/mL) and centrifuged at 13 000 G for five minutes to remove any precipitate. However, at these lower concentrations, both the Alexa-labeled and unlabeled cTn preparations remained in solution with no evidence of precipitation.

### Labeling of proteins with Alexa-N-hydroxysuccinimide (Alexa-NHS)

Purified proteins were labeled with fluorescent dye using the Alexa-N-hydroxysuccinimide (Alexa-NHS) labeling procedure described below. Pig and rat cTn preparations were labeled with Alexa Fluor 488 NHS, horse myoglobin (Myo) with Alexa Fluor 350 NHS, and pig lactate dehydrogenase (LDH) (Sigma Aldrich) with Alexa Fluor 700 NHS. First, the protein preparations were desalted on a G50 column in Buffer L (0.2 M NaCO_3_ pH 8.3, 1 M NaCl) to remove all possible traces of Tris or other amine contaminants that could otherwise interfere with the N-hydroxysuccinimide (NHS) labeling. The proteins were concentrated on ultracentrifugation columns (Amicon) with the exclusion limit of 3 K to a protein concentration of 5–10 g/L. If lower protein concentrations were used, labeling with the Alexa-NHS reagents was inefficient. Alexa Fluor 488 NHS, Alexa Fluor 350 NHS or Alexa Fluor 700 NHS (Thermo Fisher Scientific) were added in fivefold molar excess and allowed to react in Buffer L for 3–24 hours at room temperature. The reaction mixture was then supplemented with an equal volume of 2 M Tris-HCl pH 7.5 to inactivate the NHS group, and purified away from the unreacted Alexa Fluor NHS dye on a G50 column in Buffer L. High molecular weight peak fractions containing the labeled proteins were concentrated on ultracentrifugation columns (Amicon) with the exclusion limit of 3 K and stored at −20 °C. The molar ratio of the Alexa Fluor NHS label to protein was 1–3 molecules per protein. When higher levels of labeling were used, the Alexa-labeled cTn preparations had a tendency to precipitate.

### Intravenous injection of purified pig cTn complex and organ harvesting

Rats were anesthetized and reference blood samples were collected from all rats by tail snip, and tissue adhesive (Histoacryl®, B. Braun, Germany) was applied for wound closure. Intravenous (i.v.) injections of 300 µl protein cocktails containing cTn, Myo and LDH were administered via the right internal jugular vein in three rats. Blood samples (150 µl) were collected via the tail vein in conscious animals 10 min, 30 min, 60 min, 90 min, 3 h, 6 h and 24 h after the injection. Organs were harvested and frozen in liquid nitrogen before storage at −80 °C. Whole blood samples were left to stand for 60 min at room temperature, prior to centrifugation (Heraeus Fresco 17, Thermo Scientific GmbH, Germany) at 10 000 G for ten minutes at 4 °C. Serum was separated from the red blood cells and stored at −20 °C.

### Organ extract and measurement of fluorescence

Frozen organ pieces varying in weight between 0.11 g and 0.67 g from rats sacrificed after injection of fluorescent cTn, LDH and Myo were crushed using a small glass Dounce homogenizer (D8938, 2 mLSigma Aldrich, Sweden) at room temperature in an extraction buffer (phosphate buffer saline (PBS) supplemented with 10 mM EDTA and 10 mM DTT). The volume of extraction buffer was adjusted to limit the viscosity of the organ homogenate, which was a problem, especially for brain and quadriceps extracts, and the extracts were supplemented with 2% triton X-100. This was important in order to reduce turbidity in the later steps, especially in the brain and liver extracts. After extraction with gentle agitation at +8 °C for 24 h, the extracts were centrifuged at 10 000 G for 30 minutes at +4 °C. The supernatants were collected. Clear extracts (250 µl) were transferred to 96-well plates and the fluorescence was measured with a FLUOROSCAN ASCENT FL using excitation/emission (ex/em) wavelengths of 355/460 nm for Alexa Fluor 350 and ex/em wavelengths of 485/538 for Alexa Fluor 488. Alexa Fluor 700 was measured in a TECAN infinite M200 microplate reader using ex/em wavelengths of 650/750 nm. All measurements were background-subtracted using organ extracts from untreated rats. The background-subtracted fluorescence signal per gram of extracted tissue was calculated and adjusted for total organ weight in the rat^24–26^; that is, the fluorescence signal per gram of organ was adjusted with the following factors, related to the organ’s relative proportion of the total body weight in rats: liver = 1, quadriceps muscle = 0.69, brain = 0.15, lungs = 0.11, small intestine = 0.67, kidneys = 0.21, spleen = 0.06, and heart = 0.08. Relative organ retention in liver and kidneys were different from all other organs by a two-sided t-test.

### PET analysis of i.v. ^18^F-cTn injections in the rat

Pig cTn (2 mg/mL) was labeled with ^18^F for PET imaging by using an approach similar to that described by Olberg *et al*.^27^. ^18^F-cTn was then purified on a PD MidiTrap G-10 gravity column (GE Healthcare Bio-Sciences) using saline as eluent. The integrity of ^18^F-cTn was confirmed by size exclusion chromatography using a TSK gel G2000SWXL column (Tosoh Bioscience LLC) and a Thermo Scientific UltiMate 3000 Rapid Separation system. The elution pattern of ^18^F-cTn, as detected by an inline 35 µl gamma D flow cell and a Radiomatic 625TR Flow Scintillation Analyzer (PerkinElmer Inc.), was similar to that of the unlabeled cTn complex, as detected by the UltiMate™ 3000 Diode Array Detector Flow Cell (2.5 µm).

A 30-minute dynamic PET scan (small-animal PET/CT, NanoScan PC PET/CT; Mediso Ltd, Budapest, Hungary) was performed during isoflurane anesthesia after a bolus injection of about 50 µl saline containing 5 MbQ ^18^F-cTn in a lateral tail vein. The cannula was immediately flushed with 500 µl saline. The rat was then allowed to wake up and a final five-minute PET scan was performed under isoflurane anesthesia 180 minutes after a tracer injection. The following PET scan acquisition parameters were applied: Field of view, 9.6 cm in the axial direction and 10 cm in the transaxial direction, 1:5 coincidence mode and normal count rate mode. The body temperature was maintained at 37 °C throughout the whole procedure. Reconstruction of the PET data was performed with 3D OSEM and 1:3 coincidence mode, no filtering, attenuation correction (from a helical CT scan, 70 kVp, 300 ms, 360 projections reconstructed using a Shepp Logan filter), decay correction and normalization of detectors. The images were analyzed using Interview fusion software (version 2.02.029.2010 BETA).

### Cells and sample preparation

Mouse hepatoma Hepa1c1c7 cells (Sigma Aldrich 95090613) were grown in a Minimum Essential Medium Eagle (MEM) supplemented with 10% fetal bovine serum, 2 mM L-glutamine and penicillin/streptomycin. The cells were detached (trypsin-EDTA 0.05%, five minutes) and passaged twice a week. For confocal microscopy imaging the cells were plated on 8-well chamber slides (ibi-treat 8-well µ-slides from ibidi, LRI instruments Inc.) with 50 000 cells/well one day before the experiment.

### Endocytosis experiments and confocal microscopy

Cells were washed twice with serum-free medium and incubated at 4 °C or 37 °C for 30 min prior to protein addition. The cTn-Alexa Fluor 488 (10 mg/l), LDH-Alexa Fluor 488 (5 mg/L), Low-density lipoprotein (LDL) LDL-BODIPY®FL (2.5 mg/L) and Tropomyosin-Alexa Fluor 488 (2.5 mg/L) were added to each microscopy slide and the incubations continued for two hours at 4 °C in order to inhibit endocytosis, or at 37 °C to promote endocytosis. The endocytosis inhibitor Dynasore hydrate (Sigma Aldrich) (50 µM) was added to the cells 30 minutes before addition of cTn-Alexa Fluor 488. As recommended, Dynasore hydrate treatment was performed in MEM without serum. The cells were washed twice with PBS, fixed with 4% paraformaldehyde, (PFA) in PBS for 15 min at 4 °C or room temperature, and washed once again with PBS. The nuclei were stained with 1 µM DAPI in PBS for five minutes, washed 1X with PBS and 2X with MQ water, and mounted with ibidi mounting medium (LRI Instruments Inc.). Confocal imaging was performed on a Nikon Eclipse Ti confocal microscope using a Nikon Apo 60X1.40 oil immersion objective with 405 nm and/or 488 nm lasers for DAPI and Alexa Fluor 488 or BODIPY®FL, and the fluorescence was detected using the filter/bandpass of 450 nm/50 nm and 525 nm/50 nm for the blue and green channel, respectively. Images were processed using the Fiji software (previous ImageJ, free software provided by the National Institutes of Health).

### Laboratory analyses

Serum samples were stored at −20 °C before dilution in PBS supplemented with 1 g/L bovine serum albumin (BSA) and analysis at the Clinical Chemistry Laboratory at Sahlgrenska University Hospital.

Analysis of cTnT was performed using the latest versions of the Roche hs-cTnT method on Cobas and the latest versions of the Abbott hs-cTnI method on Architect with coefficients of variation (CV) below 3.3% for TnT and below 6.9% for TnI at the troponin levels analyzed in this study^18,28^.

## Results

### Organ retention of fluorescently labeled cTn

**Figure 2:**
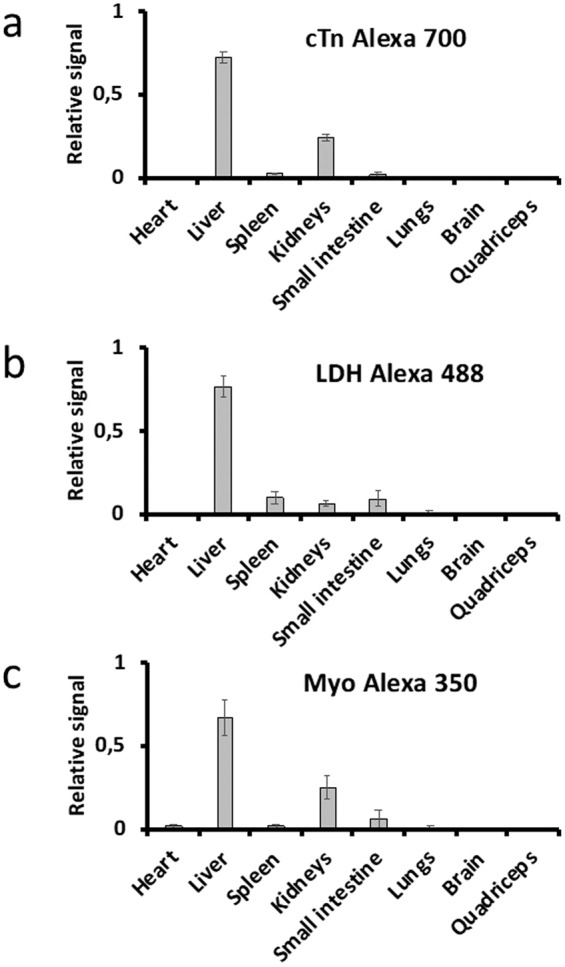
Relative organ retention of fluorescently labeled proteins. cTn (**a**), lactate dehydrogenase (LDH) (**b**) and myoglobin (Myo) (**c**) 1.5 h after a tail vein injection. Mean values from three rats. Error bars represent ±1 SD.

Rats were co-injected in the tail vein with fluorescently labeled pig cTn labeled with Alexa Fluor 488, horse myoglobin (Myo) labeled with Alexa Fluor 350, and pig lactate dehydrogenase (LDH) labeled with Alexa Fluor 700. Organs were harvested and frozen in pieces after 1.5 h, a time point when 99% of the cTnT and cTnI measured with high-sensitive clinical assays had been cleared from the blood (Fig. 1)^20^. The clearance kinetics of rat cTn and pig cTn were similar after an intravenous (i.v.) injection (Fig. 1 and Supplementary Fig. 1). Extracts were prepared from the harvested organs. The fluorescence signal in the organ extracts was measured and expressed as the relative fluorescence signal from each rat. The relative fluorescence signal from Alexa Fluor 488 was 72% in the liver, 24% in the kidneys and 2% in the spleen (Fig. 2). The organ distribution of Myo was similar to that of cTn, whereas LDH was retained mostly in the liver and only a small portion was retained in the kidneys, as previously reported^29^. The relative organ retention was similar in one rat where we performed label-switching and injected cTn labeled with Alexa Fluor 700 and LDH labeled with Alexa Fluor 488, indicating that the nature of the fluorescent dye was not important to the relative organ retention (Supplementary Fig. 3).

### Organ retention of ^18^F-labeled cTn by dynamic PET analysis

**Figure 3:**
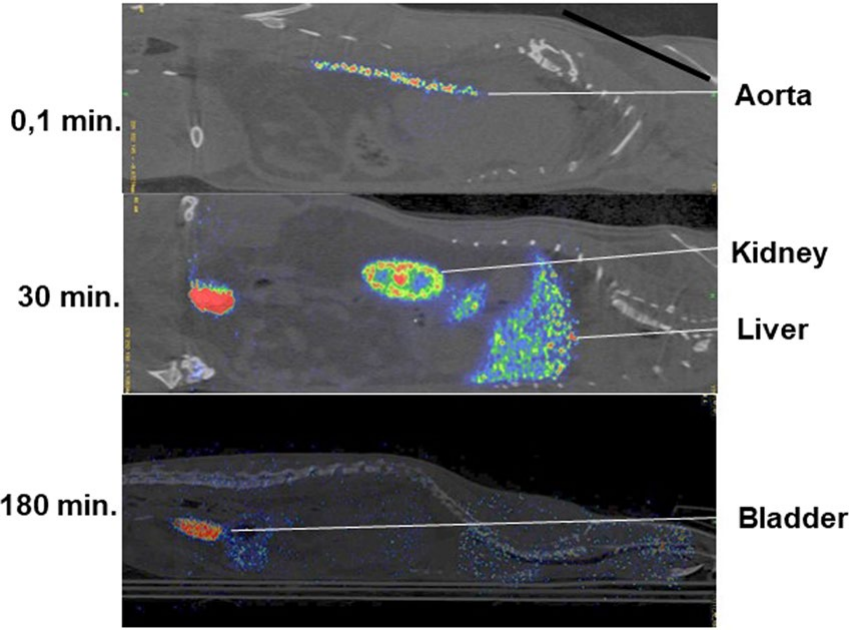
Organ retention of ^18^F-labeled pig cTn complex followed by PET. PET imaging 0.1, 30 or 180 minutes after a tail vein injection of ^18^F-labeled pig cTn complex.

**Figure 4:**
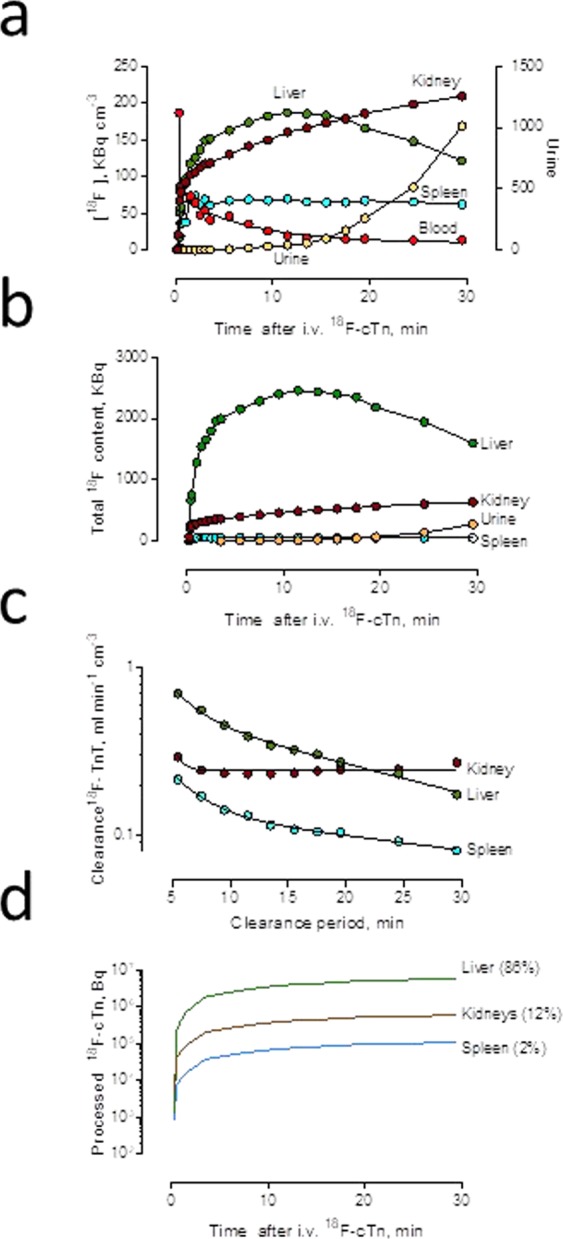
Distribution of ^18^F-cTn activity in the liver, spleen, kidneys and bladder urine, followed by PET. ^18^F-cTn activity during the first 30 min after a tail vein injection. (**a**) Concentration per cm^3^ tissue (left y axis) or bladder urine (right y axis). (**b**) Total activity in organ or bladder (**c**) Organ accumulation clearance of ^18^F-cTn calculated as organ concentration divided by the time-integrated plasma concentration of ^18^F activity for clearance periods of increasing length, from five to 30 minutes after an i.v. injection. (**d**) Total amount of ^18^F processed in organs calculated as the time-integrated product of five-minute organ accumulation clearance and plasma activity.

An ^18^F-labeled cTn preparation was injected in the tail vein in one anesthetized rat and the ^18^F signal was followed continuously by PET during a 30-minute dynamic scan (Figs. 3–4). The ^18^F activity in the blood peaked after 15 seconds and then showed a rapid decline. The ^18^F-cTn accumulated in the liver, kidneys and the spleen before ^18^F-labeled material was excreted in the urine after a delay of about 15 minutes (Fig. 4A,B). ^18^F-cTn peaked in the liver after twelve minutes, whereas the ^18^F activity continued to increase in the kidney cortex throughout the clearance period of 30 minutes. At maximum liver retention, the relative organ content of ^18^F was 82% in the liver, 16% in the kidneys and 0.4% in the bladder (Fig. 4B). The continuous accumulation of ^18^F in the renal cortex suggests that ^18^F-cTn was filtered through the glomerular membrane and retained there during the clearance period of 30 minutes (Fig. 4C).

Assuming a total organ retention of ^18^F-cTn 0–5 minutes following an i.v. injection, the accumulation clearance of cTn was calculated to be 0.70, 0.30 and 0.22 mL/min per cm^3^ tissue volume in the liver, kidney and spleen, respectively. Over extended periods, the clearance rates fell rapidly in the liver (t_½_ 17 min) and spleen (t_½_ 35 min), indicating rapid turnover in these organs (Fig. 4C). Using the 5-min clearances as representative of the organ uptake rate of ^18^F-cTn during the 30-minute clearance period, it could be predicted that the liver (volume 13.1 cm^3^), the kidneys (volume 3.0 cm^3^) and the spleen (volume 0.8 cm^3^) accounted for 86%, 12% and 2%, respectively, of the total uptake of ^18^F-cTn (Fig. 4D).

### cTn endocytosis by mouse hepatoma cells

**Figure 5:**
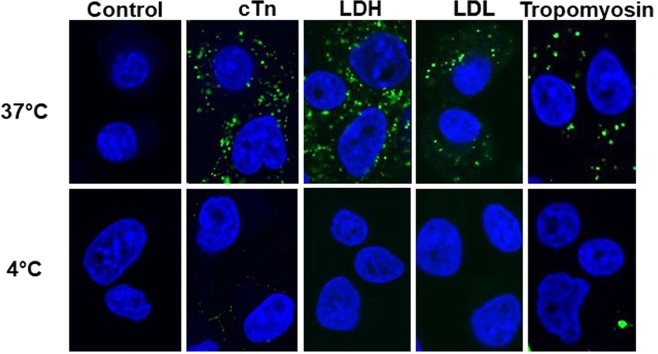
Uptake of fluorescently labeled proteins by mouse hepatoma cells. Confocal fluorescence microscopy imaging of the uptake of the Alexa Fluor 488-labeled cTn complex, lactate dehydrogenase (LDH) and tropomyosin and BODIPY®FL labeled low-density lipoprotein (LDL) by mouse hepatoma cells after 2 h incubation at 37 °C and at 4 °C. At 4 °C, the cellular uptake via endocytosis is expected to be blocked. Endocytic vesicles containing Alexa Fluor 488-labeled proteins appear as green dots. Cellular nuclei, blue, were stained with DAPI.

To examine if cTn is internalized by transformed liver cells, we exposed mouse hepatoma cells (Hepa1c1c7) to fluorescently labeled cTn, in addition to LDH, low density lipoprotein (LDL) and tropomyosin. All proteins accumulated in the cytoplasm and the uptake was blocked by incubation at +4 °C that inhibits endocytosis (Fig. 5). The cytoplasmic accumulation of cTn was lower if the cells were preincubated with the endocytosis inhibitor Dynasore (Supplementary Fig. 5). Together, these data indicate that endocytosis was involved in the uptake of cTn by transformed mouse hepatoma cells.

## Discussion

This study was inspired by our findings that rats without kidney function still had prominent clearance of cTnT^20^. Here, we show that extrarenal clearance of cTn mainly occurs in the liver. Similar accumulation of cTn in the liver and kidneys was seen if cTn was labeled with ^18^F, Alexa Fluor 488 or Alexa Fluor 700, indicating that the nature of the label did not affect the organ retention of cTn^30^. We observed a fast uptake of fluorescently labeled cTn by mouse hepatoma cells in cell culture, which was blocked by incubation at 4 °C and by the endocytosis inhibitor Dynasore and therefore likely occurs by endocytosis.

The liver removes unwanted proteins like LDH and desialylated serum proteins from plasma by receptor-mediated endocytosis^31–34^. Despite the obvious importance, surprisingly few studies have explored the clearance of cardiac damage biomarkers. Early studies indicate that myoglobin accumulated in the liver and spleen in patients who died from massive rhabdomyolysis^35,36^. Radioactive LDH is taken up by the liver in rats^35–39^. Although these studies never identified specific LDH receptors, the uptake is likely mediated by scavenger receptors, a loosely defined group of receptors that are able to bind to a plethora of unwanted proteins and direct them to endocytosis and degradation in lysosomes^40^. Mice^38,39^ and humans^29^ with mutations in scavenger receptors develop stable LDH and creatine kinase elevations, indicating that impaired scavenger receptor clearance may result in stable elevations of cell damage biomarkers also in humans.

Our previous studies have established that cTnT is cleared, in part, by glomerular filtration^20,21^, but also that low kidney function can only explain a 2–3-fold elevation of cTnT. As many patients with stable cTnT elevation due to kidney failure sometimes have levels ten times higher than expected^18^, decreased kidney clearance cannot be the only mechanism behind stable cTn elevations in patients with impaired kidney function.

The PET analysis in this study indicates that cTn was filtered through the glomerular membrane, known to allow passage of ovalbumin (43 kDa), which is similar in size to cTnT (37 kDa). Assuming a GFR of 1 mL/min per gram of kidney weight, the initial five-minute accumulation clearance of ^18^F-cTn in the renal cortex predicts a sieving coefficient of ^18^F-cTn of 0.3^41,42^. If ^18^F is injected alone in the tail vein in rats, the ^18^F activity is concentrated to the central portion of the kidney, where the collecting ducts are assembled. This is because ^18^F is not absorbed by the proximal tubules. Therefore, the fast accumulation of ^18^F activity in the renal cortex after an ^18^F-cTn injection signifies that cTn was filtered and taken up in the proximal tubuli. This explains why only very low levels of cTnT are found in the urine in patients after an MI^43^.

**Figure 6:**
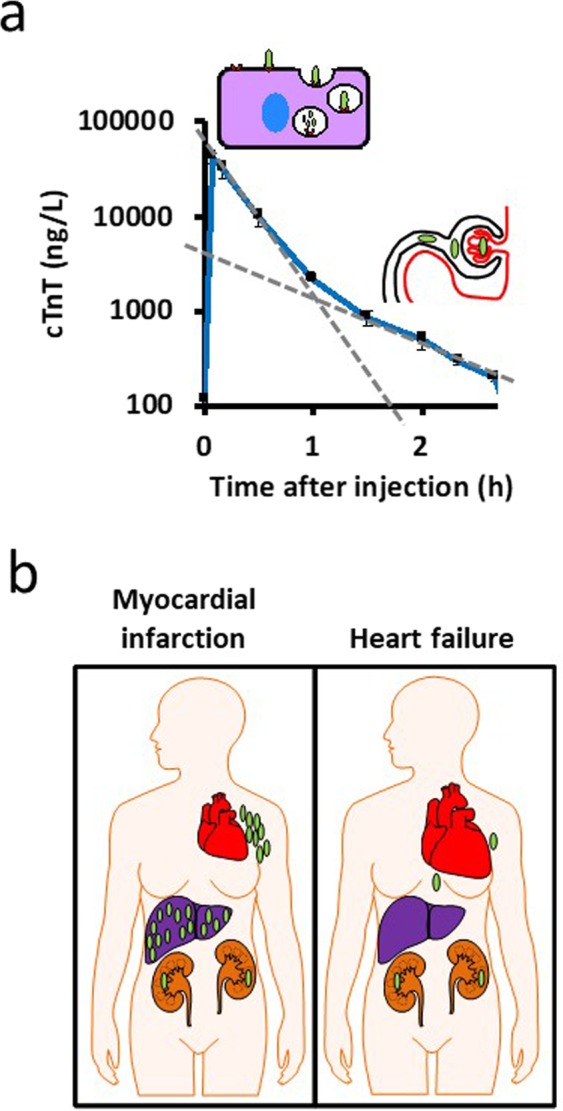
Model of cTn clearance. (**a**) Time course of the serum concentration of cTnT from Fig. 1 plotted on a log scale. There are two rates, an initial one with a half-life of 0.8 h that could be due to receptor-mediated endocytosis and degradation in the liver, and a subsequent rate with a half-life of 1.7 h that could be dominated by glomerular filtration^49^. (**b**) When cTn levels are high, such as after a myocardial infarction, cTn clearance mainly occurs by fast receptor-mediated uptake in the liver and the overall clearance of cTn would be minimally affected by slower kidney function but clear cTn with a similar half-life at both high and low levels. At the low levels of cTn observed in patients with heart failure, the levels are too low to be efficiently bound by liver receptors and slow cTn clearance via glomerular filtration becomes dominant.

It is known that receptor-mediated clearance becomes inefficient at low levels. This is well studied in the case of low-molecular weight heparin that is cleared by a fast, saturable receptor-mediated system at high levels and slow kidney-dependent clearance at low levels^44^. When heparin levels drop below the dissociation constant of the heparin receptors, receptor-mediated clearance becomes inefficient and only at these lower concentrations does the underlying, slower kidney clearance of low-molecular weight heparin become apparent^44^ (Fig. 6A).

This dual clearance model potentially explains why the clearance of cTn after a large MI is not very different in patients with or without kidney function^45,46^, whereas the low cTnT or cTnI levels found in patients with stable elevations are highly influenced by kidney function^10,47,48^.

Myoglobin (17 kDa), a protein small enough to be filtered through the glomerular membrane, is most likely also cleared by this dual system. Myoglobin accumulated in the liver and in the kidneys to a similar extent as cTn, whereas the much larger protein, LDH (150 kDa) preferentially accumulated in the liver, as previously shown^29^. Similar to cTnT, the clearance of myoglobin after an MI is not affected by kidney function^49^, whereas the low myoglobin levels found in healthy subjects are strongly linked to kidney function^50^ (Fig. 6B).

We conclude that the preferential clearance of cTn via the liver potentially explains the clinical observations that cTn clearance after a large MI is not significantly affected by the patient’s kidney function^46,51^.

However, liver clearance is probably of little importance at the low cTn levels observed in most patients with stable cTn elevations. Scavenger receptors have binding constants, the ligand concentration where half of the receptors are occupied, in the nanomolar range (Kd for the LDL receptor, 18 nmol/L^52^). The cTnT and cTnI concentrations in patients with stable elevations are at least 1000 times lower (cTnT of 100 ng/L equals 2 pmol/L). Receptor occupancy in the liver is therefore expected to be less than 0.1% at these cTnT levels, likely making liver clearance inefficient. Therefore, stable cTn elevations below 100 ng/L are probably not due to inefficient liver clearance.

Compared to humans, rats and mice with experimental myocardial infarction, where the main coronary artery has been ligated, have similar ligation-to-peak time of 10 hours^53^ but the elimination of cTn is faster in rats and mice. In humans cTn elimination after non-perfused myocardial infarction often take weeks^53^, whereas cTn is often eliminated from the circulation within 3 days after coronary artery ligation in rats and mice^54^ likely due to the small necrotic volume in mice and rats. The kinetics of cTn after myocardial infarction is a reflection of both release of cTn from necrotic cardiac tissue and its subsequent clearance from the circulation. Our studies show that cTn is slowly released due to its tight binding to insoluble thin filaments in necrotic cardiac tissue and is subjected to a “trapping effect” that makes the elution of cTn very slow if the volume of the MI is large^2,55^. As we show that cTn in the circulation has a half-life of less than one hour the kinetics of cTn after myocardial infarction^20^ is likely dominated by its slow and necrotic volume-dependent release and does not serve as a way to study cTn clearance. From our previous studies we know that cTn is cleared in the kidneys in humans^2,20^ but no other information concerning cTn clearance in humans exist.

This study has several limitations. It was performed in rats without myocardial damage. It is possible that the clearance of cTn, Myo and LDH could be different in humans undergoing myocardial infarction. Most experiments were made using cTn prepared from pig heart tissue. Although the overall clearance of cTnT and cTnI was similar after injection of purified pig or rat cTn, there could be a species-specific effect in organ targeting. Finally, it is possible that the dominant form of cTnT and cTnI that is released from damaged cardiac tissue is different from the cTn preparations used in this study. For instance, studies indicate that most cTnT is full-length during the first hour after a large MI but after a few hours, most of the circulating cTnT is made up of degradation products^56^. In fact, we see that these cTnT degradation products are cleared more slowly compared to the pig cTn complex used in this study (Muslimovic, unpublished).

In conclusion, we find that the liver and the kidneys are responsible for the clearance of cTn from plasma in the rat.

## Supplementary information

Supplementary Information.
