# Supplementary material for: The Liver and Kidneys mediate clearance of cardiac troponin in the rat
Source: Sci Rep. 2020 Apr 22;10:6791. doi: 10.1038/s41598-020-63744-8 (PMC7176693; doi:10.1038/s41598-020-63744-8)
Supplement: Supplementary file 1 — Supplementary Information. [file 41598_2020_63744_MOESM1_ESM.pdf]

Supplementary Information

The Liver and Kidneys mediate clearance of cardiac troponin in the rat

Aida Muslimovic[1#,] Vincent Fridén[1#,] Olav Tenstad[2], Karin Starnberg[1], Susanne Nyström[1], Emelie Wesén[3], Elin K. Esbjörner[3], Kristoffer Granholm[1] Bertil Lindahl[4], and Ola Hammarsten[1*]

1. Institute of Biomedicine, Department of Laboratory Medicine, Sahlgrenska University Hospital, Gothenburg University, Sweden

2. Department of Biomedicine, University of Bergen, Norway

3. Department of Biology and Biotechnology, Biology and Biological Engineering, Chemical Biology, Chalmers University of Technology, Gothenburg, Sweden

4. Department of Medical Sciences, Cardiology and Uppsala Clinical Research Center, Uppsala University, Sweden

#Equal contribution

Running title: Clearance of cardiac troponins

*Address for correspondence

Ola Hammarsten, MD, Ph.D., Professor

Senior Physician

Institute of Biomedicine

Department of Laboratory Medicine

Bruna stråket 16

Sahlgrenska Academy at the University of Gothenburg

SE-41345 Gothenburg, Sweden

Email: ola.hammarsten@clinchem.gu.se

Phone Lab: +46-31-342 1561, 342 9941

Mobile: +46-733-200834

Fax: +46-31-82 84 58

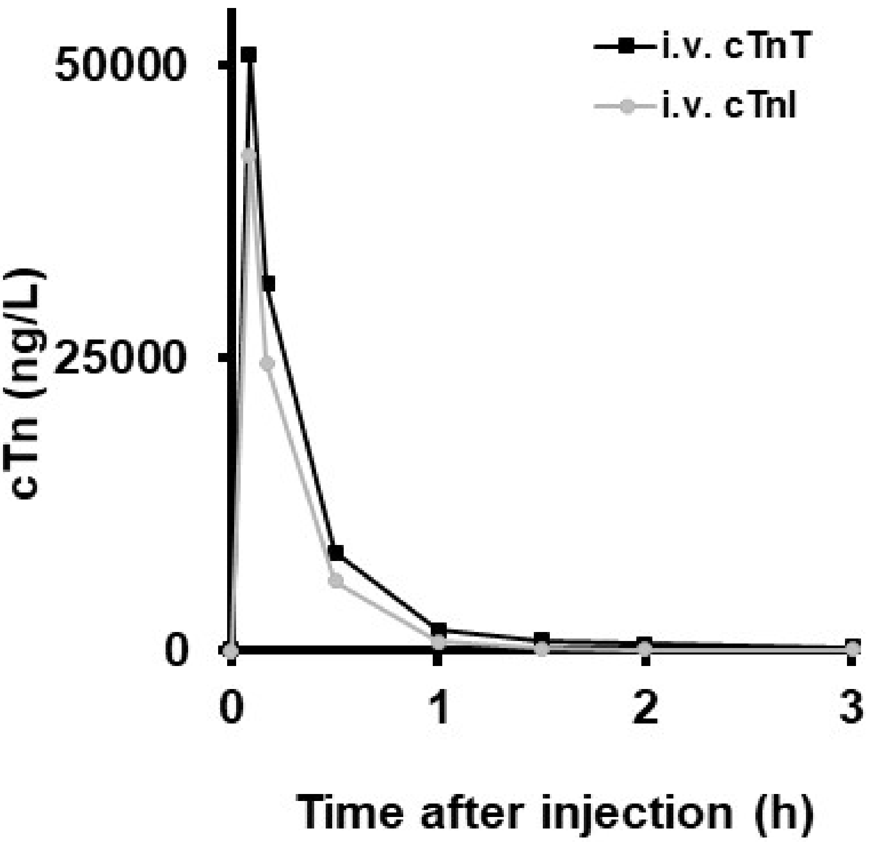

Supplementary Fig. 1 Kinetics of cTnT and cTnI clearance after jugular vein injection of purified rat cTn complex. Mean values from three rats. Error bars represent ±SD.

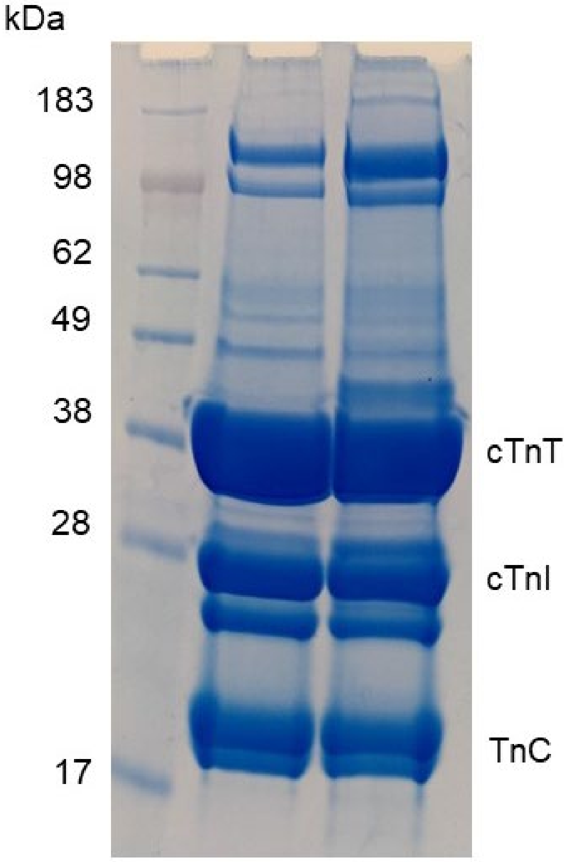

Supplementary Fig. 2 Analysis of the cTn preparation by overloaded 4-12% SDS gel stained with Coomassie blue. The positions of cardiac troponin T (cTnT), cardiac troponin I and troponin C are indicated.

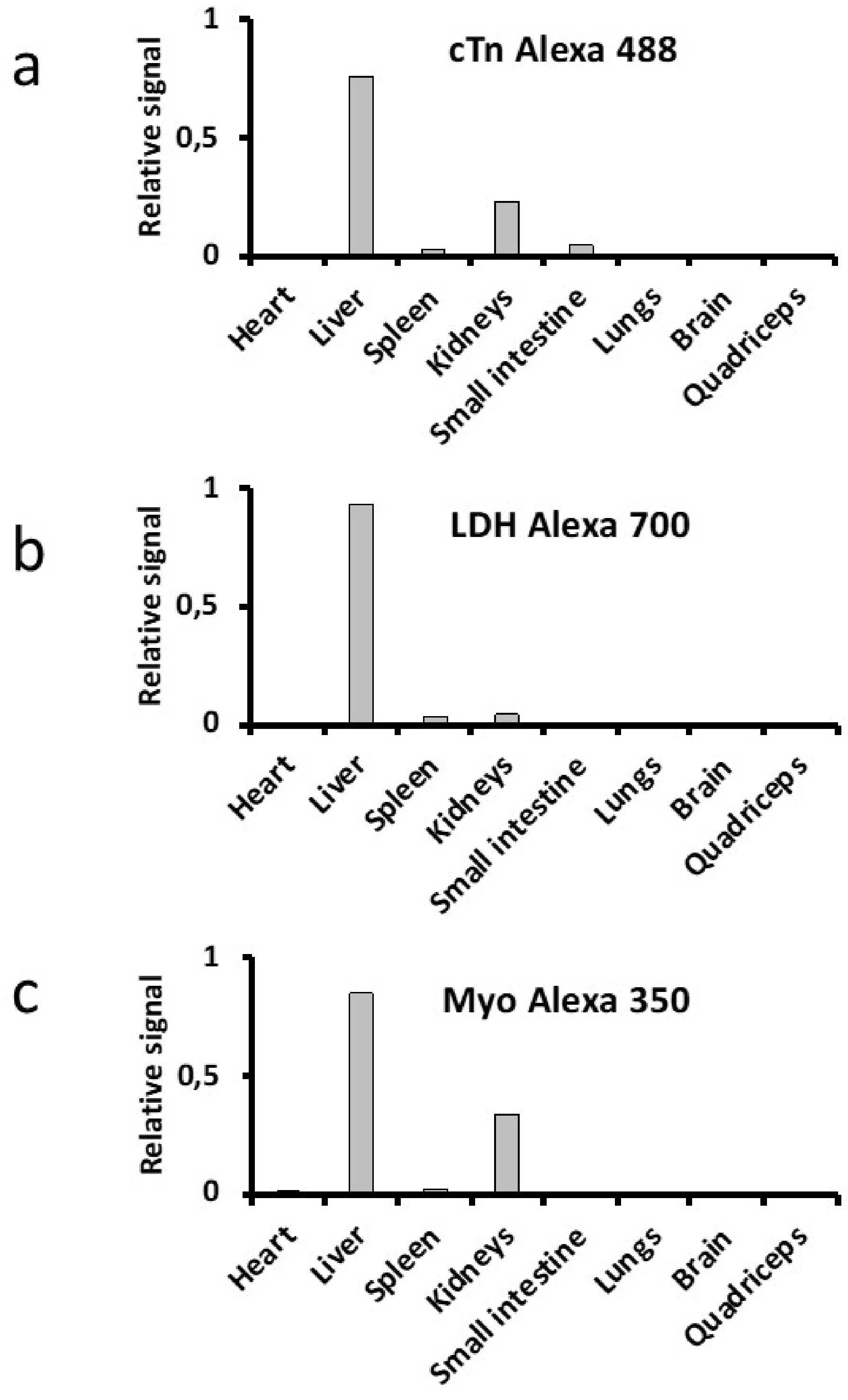

Supplementary Fig. 3 Relative organ retention of fluorescently labeled cTn (A), lactate dehydrogenase (LDH) (B) and myoglobin (Myo) (C) 1.5h after a tail vein injection in one rat.

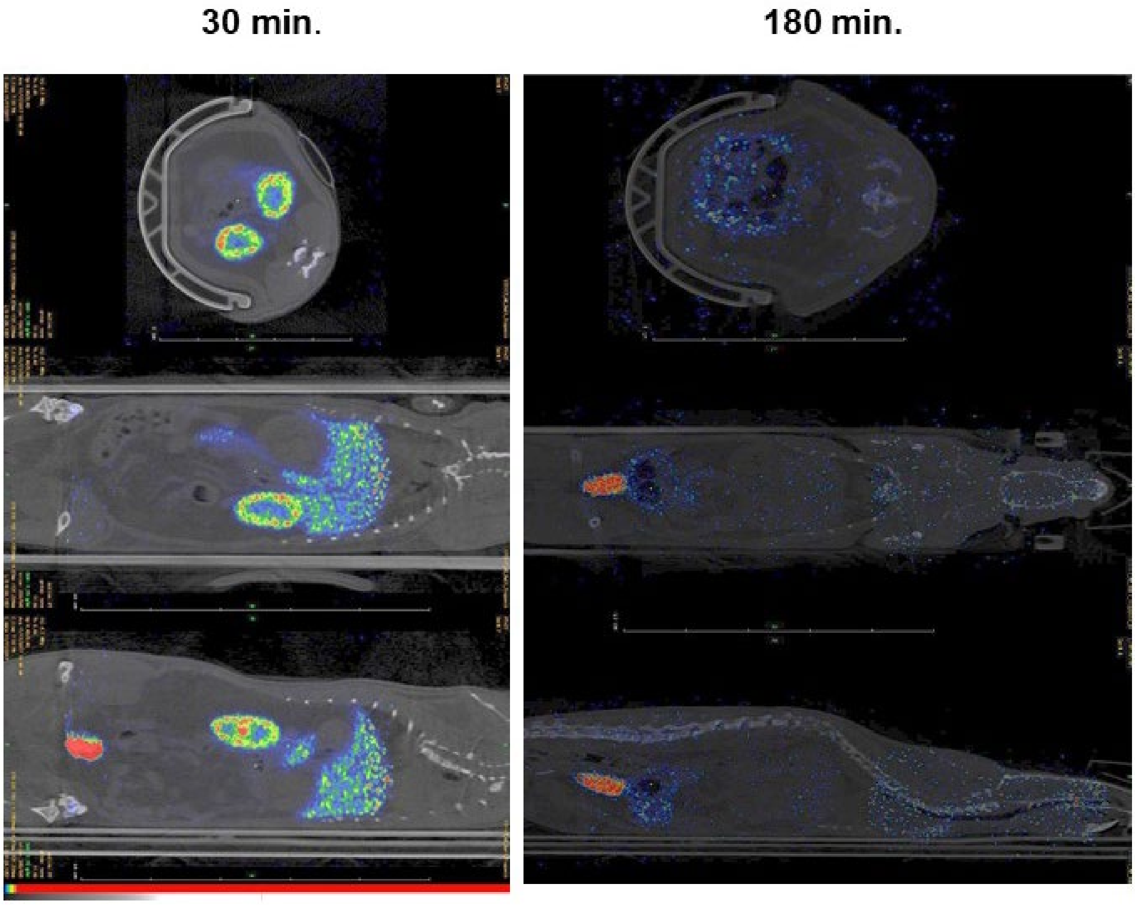

Supplementary Fig. 4 Organ retention of ^{18}F followed by PET at 30 min and 180 min after a tail vein injection of F^{18}-labeled pig cTn complex.

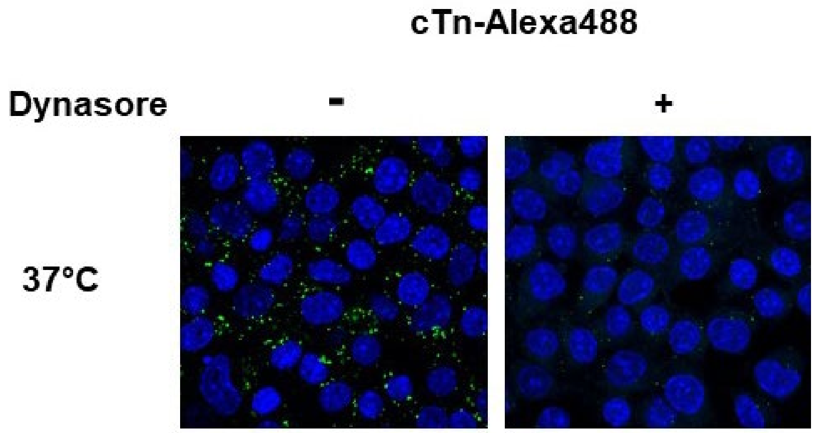

Supplementary Fig. 5 Confocal fluorescence microscopy imaging of the uptake of the Alexa 488-labeled cTn complex by mouse hepatoma Hepa1c1c7 cells preincubated (+) or not preincubated (–) 30 min with 50 mM Dynasore hydrate before addition of Alexa 488-labeled cTn complex and incubated for 2 h at 37°C. Endocytic vesicles containing Alexa 488 appear as green dots. Cellular nuclei, blue were stained with DAPI.

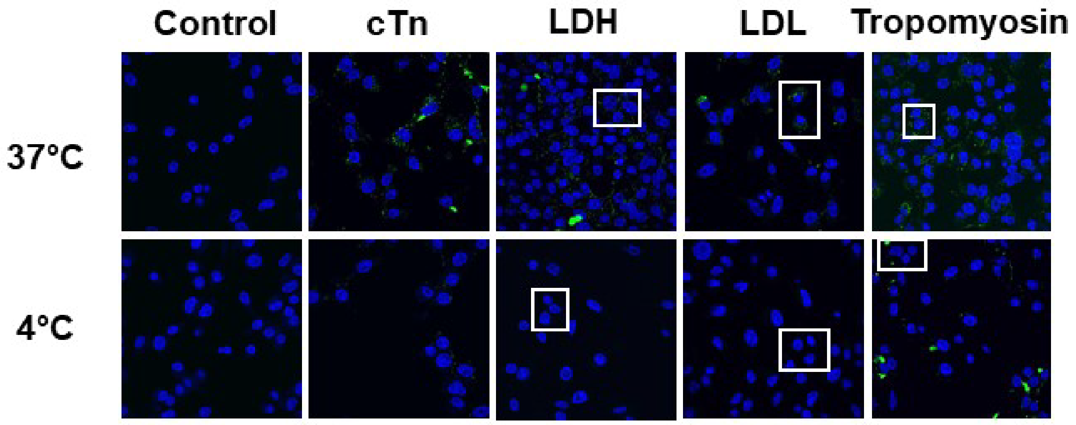

Supplementary Fig. 6 Confocal fluorescence microscopy imaging of the uptake of the Alexa 488-labeled cTn complex, lactate dehydrogenase (LDH), low-density lipoprotein (BODI PY FL-LDL) and tropomyosin by mouse hepatoma Hepa1c1c7 cells after 2 h incubation at 37°C and at 4°C. At 4°C, the cellular uptake via endocytosis is expected to be blocked. Endocytic vesicles containing Alexa 488-labeled proteins appear as green dots. Cellular nuclei, blue, were stained with DAPI. Images were taken by a Nikon Apo 60X 1.40 objective with 210.91X210.91 microns (1024X1024). White boxes represent cropped parts of the original images of LDH, LDL and Tropomyosin uptake displayed in figure 5.

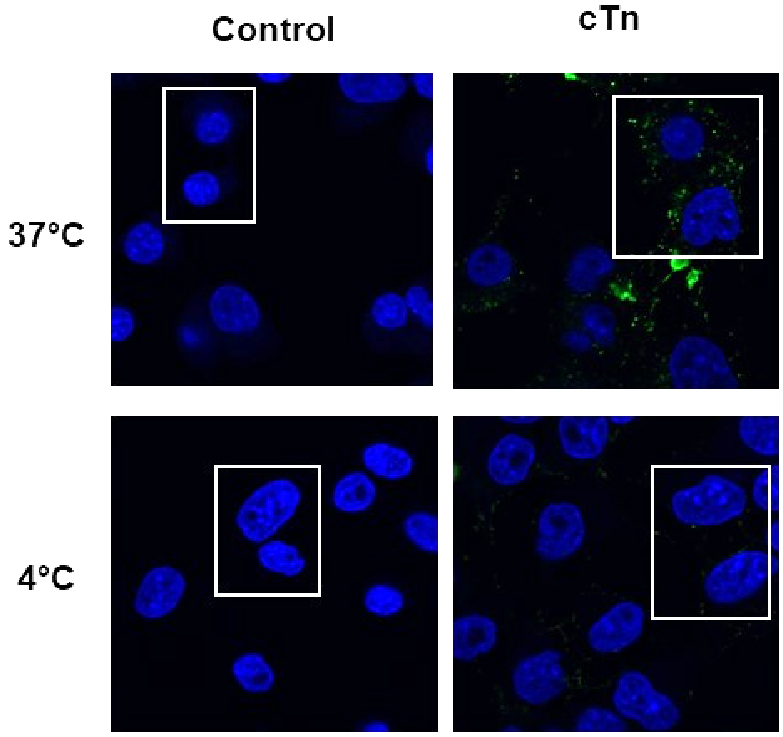

Supplementary Fig. 7 Confocal fluorescence microscopy imaging of the uptake of the Alexa 488-labeled cTn complex, by mouse hepatoma Hepa1c1c7 cells after 2 h incubation at 37°C and at 4°C. At 4°C, the cellular uptake via endocytosis is expected to be blocked. Endocytic vesicles containing Alexa 488-labeled proteins appear as green dots. Cellular nuclei, blue, were stained with DAPI. Images were taken by a Nikon Apo 60X 1.40 objective with Nyquist settings (88.36X88.36 microns (1024X1024)). White boxes represent cropped parts of the original images of cTn complex uptake displayed in figure 5.
